# Associations of alcohol intake with subclinical carotid atherosclerosis in 22,000 Chinese adults

**DOI:** 10.1016/j.atherosclerosis.2023.06.012

**Published:** 2023-06-15

**Authors:** Tianyu Zhou, Pek Kei Im, Parisa Hariri, Huaidong Du, Yu Guo, Kuang Lin, Ling Yang, Canqing Yu, Yiping Chen, Rajani Sohoni, Daniel Avery, Meiyu Guan, Meng Yang, Jun Lv, Robert Clarke, Liming Li, Robin G. Walters, Zhengming Chen, Iona Y. Millwood

**Affiliations:** 1 Clinical Trial Service Unit and Epidemiological Studies Unit (CTSU), Nuffield Department of Population Health, University of Oxford, Oxford, UK; 2 Roche Product Development, Roche, Shanghai, China; 3 Turku PET Centre, Turku University Hospital and University of Turku, Turku, Finland; 4 Medical Research Council Population Health Research Unit (MRC PHRU), Nuffield Department of Population Health, University of Oxford, Oxford, UK; 5 Chinese Academy of Medical Sciences, Beijing, China; 6 Department of Epidemiology and Biostatistics, School of Public Health, Peking University, Beijing, China; 7 Diseases Prevention and Control Department, Qingdao Cancer Hospital, Qingdao, China; 8 Department of Ultrasonography, State Key Laboratory of Complex Severe and Rare Diseases, Peking Union Medical College Hospital, Chinese Academy of Medical Sciences and Peking Union Medical College, Beijing, China

## Abstract

**Background and aims:**

We investigated the causal relevance of alcohol intake with measures of carotid artery thickness and atherosclerosis in Chinese adults.

**Methods:**

The study included 22,384 adults from the China Kadoorie Biobank, with self-reported alcohol use at baseline and resurvey, carotid artery ultrasound measurements, and genotyping data for *ALDH2-rs671* and *ADH1B-rs1229984*. Associations of carotid intima media thickness (cIMT), any carotid plaque, and total plaque burden (derived from plaque number and size) with self-reported (conventional analyses) and genotype-predicted mean alcohol intake (Mendelian randomisation) were assessed using linear and logistic regression models.

**Results:**

Overall 34.2% men and 2.1% women drank alcohol regularly at baseline. Mean cIMT was 0.70 mm in men and 0.64 mm in women, with 39.1% and 26.5% having carotid plaque, respectively. Among men, cIMT was not associated with self-reported or genotype-predicted mean alcohol intake. The risk of plaque increased significantly with self-reported intake among current drinkers (odds ratio 1.42 [95% CI 1.14-1.76] per 280 g/week), with directionally consistent findings with genotype-predicted mean intake (1.21 [0.99-1.49]). Higher alcohol intake was significantly associated with higher carotid plaque burden in both conventional (0.19 [0.10-0.28] mm higher per 280 g/week) and genetic analyses (0.09 [0.02-0.17]). Genetic findings in women suggested the association of genotype-predicted alcohol with carotid plaque burden in men was likely to due to alcohol itself, rather than pleiotropic genotypic effects.

**Conclusion:**

Higher alcohol intake was associated with a higher carotid plaque burden, but not with cIMT, providing support for a potential causal association of alcohol intake with carotid atherosclerosis.

## Graphical abstract

**Graphical Abstract:** Alcohol intake is associated with higher carotid plaque burden in Chinese men Conventional and genetic epidemiological methods were used to assess the associations of alcohol intake with cIMT, presence of carotid plaque, and carotid plaque burden, in 22,384 adults from the China Kadoorie Biobank study. Among men, higher alcohol intake was associated with higher carotid plaque burden in both the conventional and the genetic analyses.

## Introduction

Cardiovascular disease (CVD) is the leading cause of the global disease burden, affecting >500 million individuals in 2019 (1, 2). Atherosclerotic cardiovascular disease (ASCVD) has a prolonged latent period, and subclinical atherosclerosis can be present long before clinical events occur (3). Therefore, screening for subclinical arterial injury and atherosclerosis in the carotid artery is sometimes used for prediction and primary prevention of ASCVD (4, 5). Carotid intima media thickness (cIMT) and carotid plaques are routinely measured using non-invasive ultrasound examination (6), and are associated with higher risk of ASCVD (7). Globally, the prevalence of high cIMT (≥1.0 mm) and carotid plaque was 27.6% and 21.1% in 2020, respectively, an increase of about 60% for both conditions since 2000, highlighting the substantial burden of subclinical carotid arterial injury and atherosclerosis and ASCVD (8).

Alcohol consumption is a major risk factor for the global burden of disease, with an estimated 47% of adults drinking in the past year in 2017 globally, with rising alcohol use particularly among Chinese men (9). Conventional epidemiological studies have consistently reported a lower risk of CVD associated with moderate alcohol consumption compared with not drinking, however, the causality of these associations is uncertain, and the biases of reverse causality and residual confounding often affect conventional observational studies of alcohol intake (10). Recent large-scale conventional and genetic studies suggest there is no safe drinking threshold for CVD risk, but the evidence differs across CVD types (11–13). Moreover, the associations of alcohol use with subclinical carotid arterial injury and atherosclerosis remain controversial, with both positive and J-shaped associations reported in cross-sectional studies in mainly Western populations (14–22). Mendelian randomization (MR) approaches can help assess the causal effects of alcohol (23), however, large-scale MR studies assessing the causal associations of alcohol intake with cIMT or carotid plaque remain limited (24, 25).

Variants in the *ALDH2* (G>A, rs671) and *ADH1B* (G>A, rs1229984) genes alter alcohol metabolism, and can cause discomfort after drinking and strongly reduce alcohol intake (26). These variants, which are common in East Asian populations (13), can predict large differences in alcohol intake, and provide a unique opportunity to assess the causal relevance of alcohol intake for subclinical cardiovascular outcomes using an MR approach (23).

The aim of this study was to investigate the associations of cIMT and subclinical carotid atherosclerosis (presence of any carotid plaque, and total carotid plaque burden) with self-reported alcohol intake using conventional epidemiological methods, and with genotype-predicted mean alcohol intake, using data from the China Kadoorie Biobank (CKB) study.

## Materials and methods

### CKB study population

The current study was conducted in a subset of 22,384 participants from the CKB study. The CKB is a prospective cohort study which recruited 512,726 participants aged 30-79 years at baseline from five urban and five rural areas of China during 2004-2008 (27). Details of the study design and methods have been previously reported (27). The baseline survey was conducted using an interviewer-administered questionnaire which collected information on socio-demographics, medical history and major lifestyle factors, with physical measurements (e.g. blood pressure, height, weight) taken and a non-fasting blood sample collected for long-term storage. Two separate resurveys of ~5% randomly selected surviving participants were conducted in 2008 and 2013-2014, respectively, using similar procedures. The carotid ultrasound examination was conducted at the 2013-2014 resurvey (referred to as “resurvey” hereafter), for which the response rate was 76%. Ethical approval was obtained from local,national and international ethical committees. All participants provided written informed consent.

### Assessment of self-reported alcohol intake

Self-reported past and current alcohol drinking patterns were recorded using an interviewer-administered questionnaire (details in Supplementary methods), as previously described (28, 29). Participants were categorized based on their baseline alcohol drinking status into four categories: ex-drinkers; non-drinkers; occasional drinkers; and current drinkers. Current drinkers were grouped according to baseline reported alcohol intake in grams (g) per week, separately for men (<140, 140-279, 280-419, and ≥420 g/week) and women (<70 and ≥70 g/week), broadly based on the recommended cut-offs for alcohol categories by the World Health Organisation (30) and national drinking guidelines.

Self-reported alcohol consumption was re-assessed during the two resurveys using the same questionnaire. To account for regression dilution bias (31), the usual alcohol intake for baseline alcohol categories was estimated from the average of alcohol intake at the two resurveys (Table S1).

### Carotid artery ultrasonography measurements

At the resurvey, ultrasound examination was performed using a Panasonic CardioHealth Station in each of the four segments of the carotid arteries on both sides, including the distal common carotid artery (CCA), carotid bifurcation, proximal internal carotid artery, and proximal external carotid artery. The cIMT was measured only in the distal 1 cm of the CCA just before the bifurcation at 150° and 120° for right CCA and at 210° and 240° for left CCA. Mean cIMT was estimated as the mean of these four measurements. All four segments were screened for the presence of plaques. Carotid plaque was defined as any focal thickening or protrusion from the wall into the lumen with cIMT >1.5 mm (32). Carotid plaque burden was derived by standardizing the plaque number and maximum size and estimating the average, then multiplying the average value by the standard deviation (SD) of the maximum plaque thickness to provide a measure of plaque burden recorded in mm (33, 34). Details of carotid artery ultrasonography measurements were described previously (33) and in Supplementary methods.

### Genotyping and biochemistry measurements

*ALDH2-rs671* and *ADH1B*-rs1229984 were genotyped using custom Illumina Golden Gate or Affymetrix Axiom arrays at BGI, Shenzhen, among 167,734 CKB participants, which included a randomly selected subset of 151,035 participants (Supplementary methods).

Non-fasting blood samples collected at resurvey were assayed using on-site analysers for lipid measurements (including low-density lipoprotein cholesterol [LDL-C]) and glucose.

### Genotype-predicted mean alcohol intake

Using an approach described previously (13), mean alcohol intake in men was predicted using a combination of genotype and study area, both of which were strongly associated with alcohol intake. Briefly, nine genotype combinations were defined based on the genotypes for the two variants (each AA, AG, or GG): from AA/AA to GG/GG (rs671/rs1229984). Mean male alcohol intake was calculated for the combinations of the nine genotypes across the ten study areas, assigning an intake of 5 g/week to occasional drinkers and excluding ex-drinkers from the calculation. Thresholds (at cutoff points of 10, 25, 50, 100, 150 g/week) were applied to group these 90 genotype-area combinations into six categories (C1-C6) for use in genetic analyses. This allowed a reliable assessment of the shape and strength of associations with outcomes across a wide range of predicted mean male alcohol intake, while allowing adequate sample size in each category for reliable comparisons. In this way individual participants were classified only based on their genotypes and study area, but not on individual self-reported drinking patterns (which may be subject to confounding and reverse causation biases). The genetic instrument provided a measure of mean alcohol intake predicted by genotype and area, and comparisons of these six genetic categories can, where analyses are stratified by area (to adjust for any confounding by study area), be used to estimate the genotypic effects on outcomes (hence referred to as “genotype-predicted mean alcohol intake” thereafter for simplicity).

For comparison of genotypic effects by sex, women were classified into the same six categories as men based on their genotypes and study area, regardless of female drinking patterns.

### Statistical analysis

The present study included 22,384 participants who had complete data for both genetic variants, self-reported alcohol intake details at baseline and resurvey, and carotid ultrasound measurements at resurvey (Figure S1). Since alcohol consumption patterns varied substantially by sex, all analyses were conducted separately in men and women.

Mean values and percentages of selected characteristics were calculated across self-reported drinking categories. General linear models were used to estimate adjusted means and percentages of selected characteristics for individual SNP genotypes (AA, AG, GG), and for genotype-predicted mean male alcohol intake categories (C1-C6), adjusted for age, ten study areas and 12 genomic principal components (35). The associations of variables with individual SNPs were assessed by an inverse-variance-weighted meta-analysis of the per G-allele effect across ten study areas. The trend across the six genetic categories was assessed from the straight line of best fit through the adjusted mean values and their standard errors and the mean male alcohol intake of each category within each study area, which were then combined by inverse-variance-weighted meta-analysis to yield the overall area-stratified genotypic associations.

Participants with self-reported history of CVD (coronary heart disease, stroke or transient ischemic attack) at baseline or resurvey (n=2337) were excluded from conventional epidemiological analyses. Linear or logistic regression was used to assess the associations of self-reported drinking patterns with mean cIMT and carotid plaque burden, or presence of carotid plaque, adjusted for age, ten study areas, education level (no formal school; primary school; middle/high school; college/university), household income (<10,000, 10,000-34,999, 35,000+ yuan/year), and smoking status (never, ex-, occasional, current smokers) at resurvey. To correct for regression dilution bias, the means or log ORs among current drinkers were plotted against usual alcohol intake, and the slopes of the lines of best fit were described as the change in the means or log ORs per 280 g usual alcohol intake per week.

To investigate potential confounding or mediation by cardiovascular risk factors, the regression models were further adjusted for systolic blood pressure (SBP), body mass index (BMI), and LDL-C measured at resurvey as continuous variables. The associations of alcohol with carotid measurements were examined in subgroups defined by age, SBP, BMI, LDL-C, and random blood glucose level, with the trend across the slopes of best fitted lines within subgroups assessed using chi-squared trend tests. Sensitivity analyses included individuals with self-reported prior CVD.

For the genetic analyses, linear or logistic regression was used to assess the associations of genotype-predicted mean male alcohol intake with mean cIMT and carotid plaque burden, or presence of carotid plaque, without excluding participants with a history of CVD. These models were adjusted for age, study area and 12 genomic principal components. The means or log ORs were plotted against the mean male alcohol intake for each of the six genetic categories. To account for potential geographic confounding effects, the slope of the line of best fit was estimated within each study area (thus each reflecting purely genotypic effects)and combined by inverse-variance-weighted meta-analysis to yield the overall area-stratified genotypic associations, summarized as the change in the means or log ORs per 280g/week higher genotype-predicted mean male alcohol intake. Sensitivity analyses excluded those with self-reported CVD at baseline or resurvey.

Since few women consumed alcohol, any genotypic effects of the six genetic categories that are mediated by drinking alcohol should be much smaller in women than in men, but any other pleiotropic genotypic effects (i.e. genotypic effects not mediated by drinking patterns) should be similar in both sexes. Hence, similar analyses using the same six genetic categories were performed in women relating the genotypic effects in women to the genotype-predicated mean male alcohol intake in each category, to allow comparison of genotypic effects by sex.

The genotypic associations of individual genetic variants (rs671, rs1229984) with carotid artery measurements were also assessed. The age- and genomic principal components-adjusted genotypic effects (GG vs. AG genotypes) were estimated within each study area and were combined by inverse-variance-weighted meta-analysis to yield the overall area-stratified genotypic associations.

For exposure variables involving more than two categories, ORs were presented with group-specific 95% CIs calculated using “floating” standard errors to enable comparison between any two categories rather than just with the reference category (31). All analyses were conducted using SAS (Version 9.4) and figures were produced using R (version 4.0.5) and Stata/SE 16.1 (StataCorp LLC, TX, USA).

## Results

Among the 22,384 study participants, 38% (n=8503) were men and the mean age at resurvey was 60.3 (SD 10.4) years in men and 59.1 (10.0) in women (Table 1). At baseline, 34% of men and 2% of women were current drinkers (Table S2), with a corresponding prevalence of 29% and 2% at resurvey. Among both men and women, the proportion of current smokers was highest among current drinkers (Table 1). Ex-drinkers were older and had the highest mean SBP and the highest prevalence of prior CVD, especially among men (19% in ex-drinkers vs 9% in current drinkers).

Among men baseline alcohol drinking patterns varied across study areas, with the prevalence of current drinkers ranging from 11% to 57%, and mean intake among current drinkers ranging from 180 g/week to 427 g/week (Figure S2). In women, the prevalence of current drinkers was low in all study areas (<10%).

Overall the rs671 A-allele frequency was 21% (range 13% to 29% across areas), and the rs1229984 A-allele frequency was 70% (64% to 74%) (Table S3). Among men, the A-alleles for both variants were associated with lower alcohol consumption, with a stronger effect of rs671 (adjusted prevalence of current drinkers: 0.3% vs. 17% vs. 46%, AA vs. AG vs. GG) than rs1229984 (32% vs. 35% vs. 44%, AA vs. AG vs. GG) (Table S4). Among men the six genetic categories strongly predicted alcohol consumption, with a 30-fold difference in current drinking prevalence (2% vs. 61%) and a 60-fold difference in mean alcohol intake (4 vs 255 g/week) between C1 and C6 at baseline as previously described (Table 2).(12) Among women alcohol consumption levels remained low across the six genetic categories. Higher genotype-predicted mean alcohol intake was associated with higher mean SBP among men at resurvey, but was not associated with smoking or other self-reported socio-economic or lifestyle factors in either men or women (Table 2).

At resurvey, the mean cIMT was 0.70 mm among men and 0.64 mm among women, and 39% of men and 26% of women had carotid plaque (Table 1). The mean burden of carotid plaque was 0.99 mm among men and 0.65 mm among women. Among men, mean cIMT was similar across self-reported drinking categories (Table S5). Moreover, among current drinkers there was no significant dose-response association between mean cIMT and self-reported usual alcohol intake (change in cIMT [mm]: 0.001, 95% CI [-0.012, 0.013], per 280 g/week usual alcohol intake) (**Figure 2A**). For carotid plaque, the odds of having plaque was higher in ex-drinkers compared with non-drinkers, and increased in a dose-response relationship with alcohol intake amount among current drinkers, with 42% (OR=1.42; 95% CI 1.14, 1.76) higher odds per 280 g/week higher usual alcohol intake (**Figure 2B**). The association was similar in shape for carotid plaque burden, with each 280 g/week higher usual alcohol intake associated with 0.19 (95% CI 0.10, 0.28) mm higher carotid plaque burden (**Figure 2C**). The dose-response associations of carotid plaque and plaque burden were attenuated but remained significant after adjusting for SBP, and did not change materially after further adjusting for BMI, but appeared slightly stronger after further adjusting for LDL-C (Table 3).

The associations of usual alcohol intake with cIMT and carotid plaque were similar across subgroups defined by major CVD risk factors (Table S6). For carotid plaque burden, the association with usual alcohol intake tended to be stronger in men with higher SBP (p for trend=0.029), but was otherwise similar across subgroups.

Including men with prior CVD did not materially alter the associations of alcohol with carotid measurements (Table S5).

Among women, there were no clear dose-response associations of usual alcohol intake with carotid measurements, although mean cIMT and plaque burden were somewhat lower among ex-drinkers than non-drinkers (Table S7).

In genetic analyses, among men genotype-predicted mean alcohol intake was not associated with cIMT (-0.008 [95% CI -0.018, 0.003] mm per 280 g/week). Higher genotype-predicted mean alcohol intake was associated with significantly higher plaque burden (0.09 [0.02, 0.17] mm per 280 g/week) and showed a trend, although non-significant, towards higher odds of carotid plaque (1.21 [0.99, 1.49] per 280 g/week) (**Figure 2,** Table 4). Excluding men with prior CVD did not materially alter the associations (Table S8).

Among women, there were no associations between the six genetic categories and the three carotid measurements (Table 4, Table S9). The genotypic associations for carotid plaque burden were significantly different between men and women (P for heterogeneity=0.022), suggesting the effects on plaque burden in men were likely to be largely due to alcohol itself and not to pleiotropic effects of the studied genotypes.

In separate analyses of the two individual genetic variants, men with *ALDH2*-rs671 GG genotype (associated with higher alcohol intake) had a significantly higher mean carotid plaque burden (mean difference 0.05 mm, 95% CI 0.01, 0.10) compared to those with the AG genotype (Table 4). There was no association of carotid plaque burden with *ALDH2-rs671* in women (P for heterogeneity=0.010 between men and women). No differences in the three carotid measurements were observed for *ADH1B-rs1229984* genotypes in either men or women.

## Discussion

Using conventional and genetic approaches, this study assessed the causal relevance of alcohol intake with carotid artery thickness and plaque in a large Chinese population. In both conventional and genetic analyses, higher alcohol intake was associated with a higher carotid plaque burden among men. However, no clear associations were observed between alcohol intake and cIMT in either conventional or genetic analyses. Among women, very few drank alcohol and there were no associations of the genetic instruments with any of the three carotid measurements, suggesting that the genetic associations for carotid plaque burden among men were likely to be chiefly due to alcohol intake rather than pleiotropic genotypic effects.

Observational studies involving predominantly Western populations have examined the associations of alcohol intake and cIMT, with conflicting results. However, evidence from these studies were limited by relatively small sample size (N<5500) (16) (21) (36), cross-sectional study design (16, 22, 37), or failing to separate former drinkers from long-term non-drinkers (16, 22) which would introduce “sick-quitter” bias (15, 19, 21). A study involving two British cohorts (n=5403) found no differences in cIMT between non-drinkers and stable moderate drinkers but an increased cIMT among consistent heavy drinkers (21), whereas another European longitudinal study (n=3703) reported an inverse association of moderate alcohol consumption with cIMT (36). Existing studies on the relationship between alcohol drinking and carotid plaque are limited (38). With a larger sample size than previous studies, our conventional analyses in men showed overall J-shaped associations of self-reported alcohol consumption with carotid plaque and plaque burden, and clear dose-response associations of self-reported alcohol intake with carotid plaque and plaque burden among current drinkers, but no association with cIMT.

Cardiovascular risk factors, such as age, smoking, SBP, and lipid levels might confound or mediate the observed associations of alcohol intake with subclinical carotid atherosclerosis (8, 15, 16, 19, 20). We found that the association of alcohol with carotid plaque attenuated after adjusting for SBP and was stronger in men with higher SBP levels, and became slightly stronger with adjustment for LDL-C. These findings suggest that SBP and plasma lipid levels may potentially play a role in mediating or modifying the relationship between alcohol intake and subclinical atherosclerosis.

Reverse causation bias and residual confounding remain major issues even in well-designed conventional epidemiological analyses. The two genetic variants, rs671 and rs1229984, which are strongly associated with alcohol intake in East Asian populations, can be used to assess the causal effects of alcohol intake (13). The association of rs671 with subclinical carotid atherosclerosis has been assessed in two small genetic association studies each involving 300-400 East Asian men and women (24, 25). Individuals with rs671 GG genotype had higher plaque score compared with AA genotype in one study (24), but had a lower cIMT compared with AA/AG genotype in another study (25). Using a genetic instrument derived from both rs671 and rs1229984 that predicts a wide range of alcohol intake, while not associated with conventional confounders such as smoking, we have previously reported a causal association of alcohol drinking with stroke risk (13). In the present study we extended the investigation into carotid atherosclerosis and found a significant, although modest, dose-response relationship between genotype-predicted mean alcohol intake and carotid plaque burden, which corroborated the conventional analyses, with directionally consistent genetic associations with presence of carotid plaque, but no associations with cIMT. Our analyses of individual variants showed that the association between genotype-predicted mean alcohol intake and carotid plaque burden was likely to be mainly driven by rs671, which may reflect the stronger influence on alcohol intake of rs671 compared with rs1229984. The null genetic findings in women, who rarely drank alcohol despite their genotypes, provide further support for the associations for carotid plaque burden seen in men being potentially due to the causal effects of alcohol intake.

Previous analyses in CKB only showed moderate correlation between cIMT and carotid plaque burden (correlation coefficient 0.51), with somewhat weaker correlation in individuals with carotid plaque (correlation coefficient 0.36) (33, 34). Although cIMT and carotid plaque share similar risk factors (8, 39), the natural history and clinical significance of these measurements differ. Carotid plaque is considered to be an indicator of early atherosclerotic disease (40), and compared to cIMT, may be more strongly influenced by levels of SBP and diabetes (41, 42). The differing associations of alcohol with cIMT and with carotid plaque in the present study may reflect the distinct role of cIMT from carotid plaque in carotid atherosclerosis. Nevertheless, the precise mechanisms through which alcohol may influence different aspects of carotid arterial injury and atherosclerosis are not fully understood and further investigations are warranted to understand the underlying pathophysiological mechanisms.

This is the first genetic epidemiological study using rs671 and rs1229984 in addition to self-reported drinking patterns to assess the causal associations between alcohol intake and three different carotid artery measurements in a large Chinese population. In the present analyses, ex-drinkers could be distinguished from others based on information acquired for past drinking, and repeated alcohol measurements allowed us to estimate usual alcohol intake over an 8-year period and account for regression dilution bias in our analyses. Furthermore, with a strong genetic instrument that predicted a wide range of alcohol intake in men, and the ability to assess potential pleiotropy among women who drank little alcohol, the study was able to assess potential casual relationships. However, our study also had several limitations. First, the periodic resurvey can only include surviving participants and the response rate among eligible surviving participants was around 80%, suggesting that survival bias might exist and those who had a poor health status and very heavy/problem drinkers might have been reluctant or unable to attend the resurvey, leading to a potential underestimation of the associations. Second, our study only had carotid artery measurements at one time point and was unable to assess progression of subclinical atherosclerosis. In future investigations, measurements of progression of cIMT and carotid plaque would allow the relationships between alcohol intake and carotid aterial injury and subclinical atherosclerosis, and with CVD outcomes, to be more thoroughly explored. Third, although our study was the largest to date using a strong genetic instrument, it was limited to a subset of the CKB population. Future well-powered genetic epidemiological studies are warranted to further clarify the causal effects of alcohol on cIMT and carotid plaque.

In summary, genetic epidemiological analyses suggest that alcohol consumption may be causally associated with a higher burden of carotid plaque but not with cIMT in this Chinese population. The findings of the present study provide no evidence for a causal protective effect of moderate alcohol intake on carotid atherosclerosis. This study provides evidence to support the strategy of lowering alcohol consumption to prevent atherosclerosis and subsequent ASCVD.

## Supplementary Material

Supplementary file

## Figures and Tables

**Figure 1:**
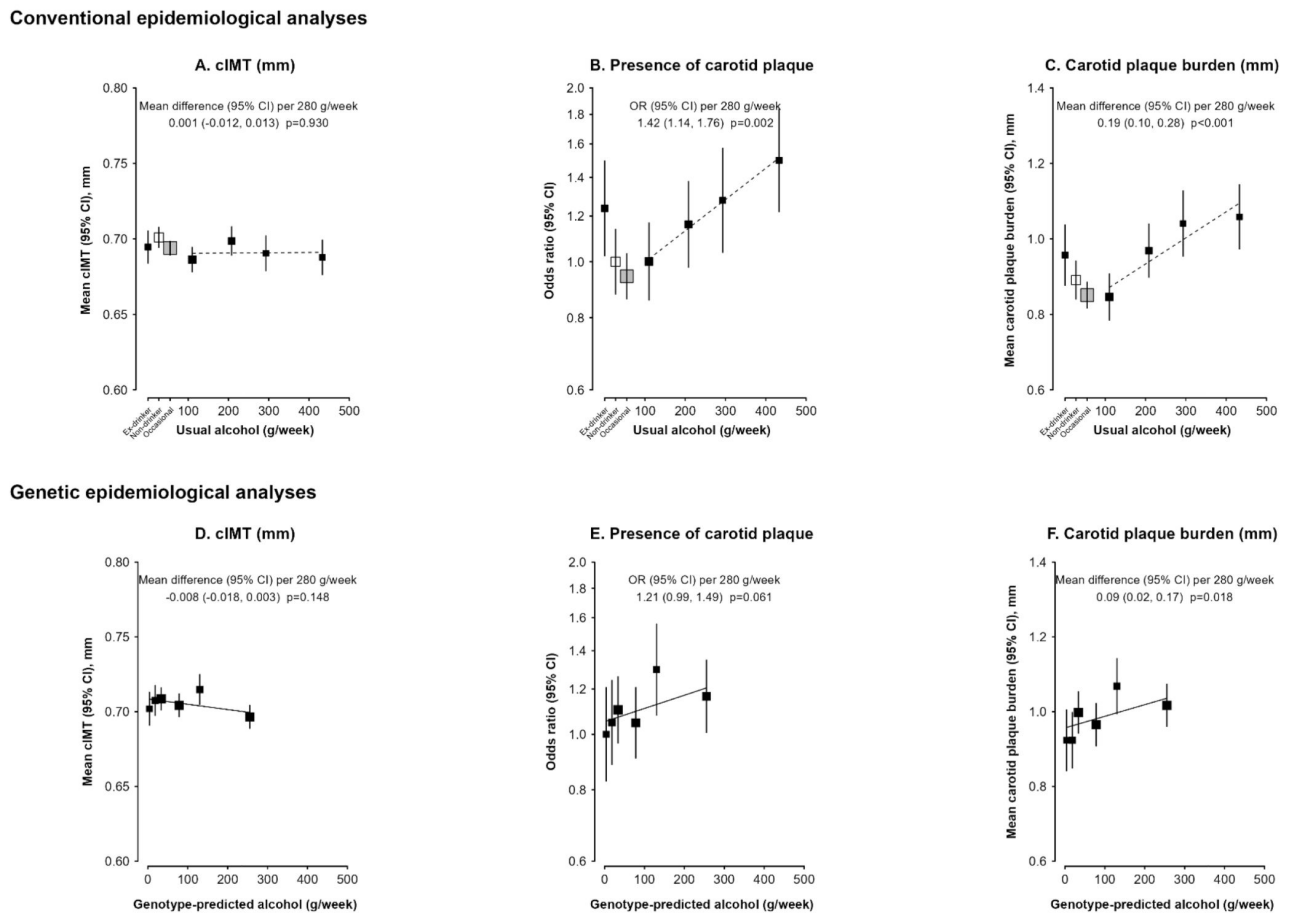
Associations of carotid measurements with self-reported alcohol consumption and with genotype-predicted mean alcohol intake, in men Conventional epidemiological analyses (A–C) of baseline self-reported drinking patterns with cIMT (A), presence of carotid plaque (B), and carotid plaque burden (C) in men without prior cardiovascular disease. The reference group was non-drinkers and results were adjusted for age, area, education, income, and smoking. The means or odds ratios for current drinkers were plotted against usual alcohol intake, with a fitted line giving the mean change or odds ratio (95% CI) per 280 g intake per week usual alcohol intake. Genetic epidemiological analyses (D–F) of genotype predicted mean alcohol intake with cIMT (D), presence of carotid plaque (E), and carotid plaque burden (F) in all men. Results were adjusted for age, area, and genomic principal components. The means or odds ratios were plotted against genotype-predicted mean alcohol intake, with the mean change or odds ratio (95% CI) per 280 g intake per week genotype-predicted mean alcohol intake calculated within study areas and combined by inverse variance-weighted meta-analysis. The area of each square is inversely proportional to the variance of the least square mean in (A, C, D, F), and the variance of the log odds in (B, E). The group-specific 95% CIs, calculated from this variance, are shown by error bars. cIMT: carotid intima media thickness; CI: confidence interval.

**Table 1:** Characteristics of men and women at resurvey by self-reported baseline alcohol drinking categories

	Overall	Ex-drinkers	Non-drinkers	Occasional drinkers	Current drinkers
**Men**	(n=8503)	(n=680)	(n=1654)	(n=3264)	(n=2905)
Age (SD), year	60.3 (10.4)	63.9 (9.7)	63.6 (10.6)	58.9 (10.4)	59.2 (9.9)
Education ≥ 6 years, %	57.3	51.0	44.6	64.0	58.5
Household income ≥ 35000 RMB/year, %	62.6	58.8	60.7	60.0	67.5
Prior CVD history^a^, %	10.9	19.1	11.7	10.2	9.2
Current smokers, %	50.5	48.1	43.3	47.2	59.0
SBP (SD), mmHg	137.0 (19.7)	141.0 (21.9)	137.4 (20.6)	135.0 (19.0)	138.1 (19.2)
BMI (SD), kg/m^2^	24.0 (3.4)	24.1 (3.4)	23.3 (3.5)	24.1 (3.3)	24.2 (3.4)
Physical activity (SD), MET-h/day	18.8 (15.0)	15.5 (13.6)	17.5 (15.2)	19.9 (15.8)	19.2 (14.3)
cIMT, mm (SD)	0.70 (0.16)	0.73 (0.16)	0.73 (0.17)	0.70 (0.16)	0.69 (0.15)
Carotid plaque, %	39.1	48.8	40.3	36.3	39.3
Carotid plaque burden, mm (SD)	0.99 (1.19)	1.24 (1.28)	1.05 (1.22)	0.92 (1.16)	0.98 (1.18)
**Women**	(n=13881)	(n=125)	(n=9019)	(n=4442)	(n=295)
Age (SD), year	59.1 (10.0)	64.6 (9.3)	59.9 (10.1)	57.4 (9.5)	60.9 (10.3)
Education ≥ 6 years, %	41.5	38.4	34.8	55.4	39.0
Household income ≥ 35000 RMB/year, %	58.3	46.4	61.0	53.9	49.2
Prior CVD history*, %	10.2	16.8	9.8	10.6	11.9
Current smokers, %	1.5	6.4	1.0	1.6	14.2
SBP (SD), mmHg	136.5 (21.3)	139.6 (23.8)	137.6 (21.4)	134.1 (20.6)	136.5 (21.7)
BMI (SD), kg/m^2^	24.3 (3.5)	24.2 (3.5)	24.1 (3.5)	24.6 (3.5)	23.9 (3.7)
Physical activity (SD), MET-h/day	17.6 (12.8)	14.5 (10.1)	17.6 (13.4)	17.6 (11.6)	17.4 (11.3)
cIMT, mm (SD)	0.64 (0.13)	0.64 (0.13)	0.65 (0.14)	0.64 (0.13)	0.64 (0.12)
Carotid plaque, %	26.5	28.0	26.2	26.7	28.8
Carotid plaque burden, mm (SD)	0.65 (0.99)	0.62 (1.00)	0.65 (0.99)	0.66 (0.99)	0.71 (1.01)

Abbreviations: RMB: renminbi; CVD: cardiovascular disease; SBP: systolic blood pressure; BMI: body mass index; MET-h/day: metabolic equivalents of task per hour per day; cIMT: carotid intima media thickness; SD: standard deviation.

a Self-reported CVD history at baseline or resurvey.

**Table 2:** Characteristics at resurvey, and drinking patterns, by categories of genotype and study area, in men and women

	Genotype-area categories						P_trend_^a^
	C1	C2	C3	C4	C5	C6	
**Men**				
Drinking patterns^b^	(n=4269)	(n=6353)	(n=11974)	(n=13527)	(n=9047)	(n=15814)	
Current drinkers^c^, %	1.7	10.6	15.0	30.2	48.5	61.4	<0.001
Mean alcohol intake^c^ (SD), g/week	4.0 (28.5)	18.3 (65.3)	33.5 (108.1)	78.3 (160.9)	130.2 (190.6)	255.5 (278.7)	<0.001
Resurvey characteristics^c^	(n=620)	(n=991)	(n=1652)	(n=2016)	(n=1209)	(n=2015)	
Age (SD), year	60.1 (10.2)	60.0 (10.6)	60.2 (10.4)	60.7 (10.6)	59.8 (10.7)	60.5 (10.0)	0.543
Education ≥ 6 years, %	56.6	54.4	55.8	56.9	61.4	58.0	0.077
Household income ≥ 35000 RMB/year, %	61.3	64.3	63.1	61.6	63.5	62.1	0.913
Prior CVD history^d^, %	10.7	10.4	10.6	11.6	12.1	9.9	0.877
Current smokers, %	46.2	50.0	51.0	49.4	53.5	51.0	0.145
SBP (SD), mmHg	135.0 (20.2)	135.9 (20.1)	135.9 (19.2)	137.3 (19.5)	138.4 (20.3)	138.0 (19.1)	<0.001
BMI (SD), kg/m^2^	23.9 (3.5)	23.9 (3.3)	23.9 (3.4)	24.0 (3.4)	23.9 (3.5)	24.2 (3.3)	0.060
Physical activity (SD), MET-h/day	18.9 (16.3)	18.6 (15.8)	18.6 (15.5)	18.9 (14.5)	19.7 (13.9)	18.6 (14.8)	0.946
**Women**				
Drinking patterns^b^	(n=6439)	(n=9723)	(n=17174)	(n=19943)	(n=13051)	(n=23721)	
Current drinkers, %	0.1	0.5	0.5	1.5	3.5	4.0	<0.001
Mean alcohol intake (SD), g/week	0.6 (2.6)	1.9 (3.9)	1.2 (6.1)	3.5 (13.4)	5.4 (23.0)	7.8 (41.7)	<0.001
Resurvey characteristics^c^	(n=1036)	(n=1636)	(n=2557)	(n=3295)	(n=1703)	(n=3654)	
Age (SD), year	59.5 (10.2)	59.5 (9.9)	58.8 (10.0)	58.9 (9.8)	58.9 (10.4)	59.5 (9.9)	0.181
Education > 6 years, %	43.1	42.6	40.7	41.4	40.3	41.9	0.403
Household income > 35000 RMB/year, %	58.6	58.9	60.0	56.6	60.5	57.4	0.330
Prior CVD history^d^, %	11.0	9.5	9.6	11.1	9.5	10.2	0.782
Current smokers, %	1.2	1.1	1.7	1.3	1.5	1.7	0.885
SBP (SD), mmHg	136.9 (21.7)	136.3 (21.3)	137.3 (21.4)	136.4 (21.4)	136.0 (22.1)	136.2 (20.3)	0.244
BMI (SD), kg/m^2^	24.3 (3.5)	24.2 (3.5)	24.2 (3.4)	24.3 (3.7)	24.1 (3.5)	24.4 (3.6)	0.153
Physical activity (SD), MET-h/day	16.8 (13.0)	16.8 (11.7)	18.0 (14.5)	17.7 (11.5)	18.1 (12.2)	17.6 (13.0)	0.121

RMB: renminbi; CVD: cardiovascular disease; SBP: systolic blood pressure; BMI: body mass index; MET-h/d: metabolic equivalents of task per hour per day; SD: standard deviation.

a P-trend is from the straight line of best fit through the age- and genomic principal component-adjusted mean values and their standard errors and the mean male alcohol intake across the six genetic categories within each study area, which were then combined by inversevariance-weighted meta-analysis to yield the overall area-stratified genotypic associations;

b Prevalence of current drinkers and mean alcohol intake at baseline were unadjusted and were calculated in a sample of 60,984 men and 90,051 women in CKB with genotype information as previously described (Millwood et al 2019 Lancet 393:1831-1842) The genetic instrument strength in men was F-statistic 1752 (range by area 43-783), variance in alcohol intake explained (r^2^) 13.6% (1.2%-22.5%);

c Means and prevalences of resurvey characteristics were adjusted for age, study area and genomic principal components as appropriate;

d Self-reported CVD history at baseline or resurvey.

**Table 3:** Adjusted associations of carotid measurements with self-reported alcohol intake in male current drinkers

Male current drinkers, n=2905	Effect per 280 g/week usual alcohol (95% CI)	P-value
cIMT, mm		
Main model	0.001 (-0.012, 0.013)	0.930
+SBP	-0.006 (-0.018, 0.006)	0.340
+SBP and BMI	-0.006 (-0.017, 0.006)	0.361
+SBP, BMI and LDL-C	-0.002 (-0.015, 0.012)	0.799
Main model including prior CVD	0.002 (-0.015, 0.010)	0.696
Carotid plaque (OR)		
Main model	1.42 (1.14, 1.76)	0.002
+SBP	1.32 (1.06, 1.64)	0.013
+SBP and BMI	1.32 (1.06, 1.64)	0.013
+SBP, BMI and LDL-C	1.49 (1.16, 1.91)	0.002
Main model including prior CVD	1.37 (1.11, 1.68)	0.003
Carotid plaque burden, mm		
Main model	0.19 (0.10, 0.28)	<0.001
+SBP	0.15 (0.07, 0.24)	<0.001
+SBP and BMI	0.15 (0.06, 0.24)	<0.001
+SBP, BMI and LDL-C	0.20 (0.10, 0.30)	<0.001
Main model including prior CVD	0.18 (0.09, 0.27)	<0.001

Main model was adjusted for age, area, education, household income, and smoking, and participants with self-reported history of cardiovascular disease at baseline or resurvey were excluded. Participants with missing LDLC data were excluded from models adjusted for LDL-C. cIMT: carotid intima media thickness; SBP: systolic blood pressure; BMI: body mass index; LDL-C: low-density lipoprotein cholesterol; OR: odds ratio; CI: confidence interval

**Table 4:** Associations of carotid measurements with genotype-predicted alcohol intake, and with *ALDH2-rs671* and *ADH1B-rs1229984* genotypes, in men and women

	Effect per 280 g/week genotype-predicted mean male^a^ alcohol intake (95% CI)	P-value	P_het_^b^	*ALDH2*-rs671 GG vs AG Effect (95% CI)	P-value	P_het_^b^	*ADH1B*-rs1229984 GG vs AG Effect (95% CI)	P-value	P_het_^b^
cIMT, mm									
Men	-0.008 (-0.018, 0.003)	0.148		-0.002 (-0.009, 0.004)	0.508		0.003 (-0.008, 0.013)	0.631	
Women	-0.004 (-0.011, 0.002)	0.186	0.589	-0.004 (-0.008, 0.001)	0.090	0.738	0.004 (-0.003, 0.011)	0.267	0.855
Carotid plaque, OR									
Men	1.21 (0.99, 1.49)	0.061		1.03 (0.92, 1.16)	0.573		1.06 (0.87, 1.28)	0.572	
Women	0.98 (0.81, 1.17)	0.806	0.120	0.93 (0.84, 1.03)	0.160	0.178	0.97 (0.83, 1.15)	0.740	0.518
Carotid plaque burden, mm									
Men	0.09 (0.02, 0.17)	0.018		0.05 (0.01, 0.10)	0.027		-0.02 (-0.10, 0.06)	0.591	
Women	-0.01 (-0.06, 0.03)	0.600	0.022	-0.02 (-0.05, 0.01)	0.195	0.010	-0.03 (-0.08, 0.02)	0.247	0.883

Analyses were adjusted for age, and genomic principal components within each study area, and then combined by inverse-variance-weighted meta-analysis to yield the overall area-stratified genetic associations. The genetic instrument strength was assessed in 60,984 men with genotype information: Main genetic instrument F-statistic 1752 (range by area 43-783), variance in alcohol intake explained (r^2^) 13.6% (1.2%-22.5%); *ALDH2-rs671* F-statistic 3267 (31–891), r^2^ 10.5% (1.0%-22.6%); ADH1B-rs1229984 F-statistic 191 (4–43), r^2^ 0.7% (0.1%-1.4%).

a As women consumed little alcohol, the same six genotypic-area categories in women were used to estimate the genotypic effects in the same way as in men, in order to evaluate potential pleiotropic effects by comparing effects in men (who drank alcohol) with women.

b P-value for heterogeneity between men and women.

## Data Availability

The China Kadoorie Biobank (CKB) is a global resource for the investigation of lifestyle, environmental, blood biochemical and genetic factors as determinants of common diseases. The CKB study group is committed to making the cohort data available to the scientific community in China, the UK and worldwide to advance knowledge about the causes, prevention and treatment of disease. For detailed information on what data is currently available to open access users and how to apply for it, visit: https://www.ckbiobank.org/data-access. Researchers who are interested in obtaining the raw data from the China Kadoorie Biobank study that underlines this paper should contact ckbaccess@ndph.ox.ac.uk. A research proposal will be requested to ensure that any analysis is performed by bona fide researchers and - where data is not currently available to open access researchers - is restricted to the topic covered in this paper.

## Non-standard Abbreviations and Acronyms

ADH1B: alcohol dehydrogenase 1B
ALDH2: aldehyde dehydrogenase 2
ASCVD: atherosclerotic cardiovascular disease
BMI: body mass index
CCA: common carotid artery
CI: confidence interval
cIMT: carotid intima media thickness
CKB: China Kadoorie Biobank
CVD: cardiovascular disease
LDL-C: low-density lipoprotein cholesterol
MR: Mendelian randomization
OR: odds ratio
SD: standard deviation
SBP: systolic blood pressure
