# Supplementary material for: Associations of alcohol intake with subclinical carotid atherosclerosis in 22,000 Chinese adults
Source: Atherosclerosis. Author manuscript; Available in PMC 2023 Sep 13. (PMC7615083; doi:10.1016/j.atherosclerosis.2023.06.012)
Supplement: Supplementary file [file EMS187522-supplement-Supplementary_file.docx]

**Supplementary materials**

**Associations of alcohol intake with subclinical carotid atherosclerosis in 22,000 Chinese adults**

Tianyu Zhou, Pek Kei Im, Parisa Hariri, Huaidong Du, Yu Guo, Kuang Lin, Ling Yang, Canqing Yu, Yiping Chen, Rajani Sohoni, Daniel Avery, Meiyu Guan, Meng Yan, Jun Lv, Robert Clarke, Liming Li, Robin G. Walters, Zhengming Chen, Iona Y. Millwood, China Kadoorie Biobank Collaborative Group

**Table of contents**

**Page**

2 Members of the China Kadoorie Biobank Collaborative Group

3 Supplementary methods

6 Figure S1. Participant flow chart

7 Table S1 Self-reported alcohol intake at baseline and resurveys by baseline defined alcohol intake groups, in men and women

8 Table S2. Characteristics of men and women at resurvey

9 Figure S2. Proportion of current drinkers, and mean alcohol intake among current drinkers, in men and women by study areas

10 Table S3. Allele frequency of ADH1B-rs1229984 G>A and ALDH2-rs671 G>A, by study areas

11 Table S4. Alcohol drinking patterns by ALDH2-rs671 and ADH1B-rs1229984 genotypes, in men and women

12 Table S5. Associations of carotid measurements with self-reported alcohol consumption in men

13 Table S6. Associations of carotid measurements with self-reported alcohol intake, among subgroups in male current drinkers

14 Table S7. Associations of carotid measurements with self-reported alcohol consumption in women

15 Table S8. Associations of carotid measurements with genotype-predicted mean alcohol intake in men

16 Table S9. Associations of carotid measurements with six genotypic categories, in women

**Members of the China Kadoorie Biobank Collaborative Group**

**International Steering Committee:** Junshi Chen, Zhengming Chen (PI), Robert Clarke, Rory Collins, Yu Guo, Liming Li (PI), Chen Wang, Jun Lv, Richard Peto, Robin Walters.

**International Co-ordinating Centre, Oxford:** Daniel Avery, Maxim Barnard, Derrick Bennett, Ruth Boxall, Sushila Burgess, Ka Hung Chan, Yiping Chen, Zhengming Chen, Johnathan Clarke, Robert Clarke, Huaidong Du, Ahmed Edris Mohamed, Hannah Fry, Simon Gilbert, Pek Kei Im, Andri Iona, Maria Kakkoura, Christiana Kartsonaki, Hubert Lam, Kuang Lin, James Liu, Mohsen Mazidi, Iona Millwood, Sam Morris, Qunhua Nie, Alfred Pozarickij, Paul Ryder, Saredo Said, Dan Schmidt, Becky Stevens, Iain Turnbull, Robin Walters, Baihan Wang, Lin Wang, Neil Wright, Ling Yang, Xiaoming Yang, Pang Yao.

**National Co-ordinating Centre, Beijing:** Yu Guo, Xiao Han, Can Hou, Qingmei Xia, Chao Liu, Jun Lv, Pei Pei, Dianjianyi Sun, Canqing Yu.

**10 Regional Co-ordinating Centres:**

**Guangxi** Provincial CDC: Naying Chen, Duo Liu, Zhenzhu Tang. Liuzhou CDC: Ningyu Chen, Qilian Jiang, Jian Lan, Mingqiang Li, Yun Liu, Fanwen Meng, Jinhuai Meng, Rong Pan, Yulu Qin, Ping Wang, Sisi Wang, Liuping Wei, Liyuan Zhou. **Gansu** Provincial CDC: Caixia Dong, Pengfei Ge, Xiaolan Ren. Maiji CDC: Zhongxiao Li, Enke Mao, Tao Wang, Hui Zhang, Xi Zhang. **Hainan** Provincial CDC: Jinyan Chen, Ximin Hu, Xiaohuan Wang. Meilan CDC: Zhendong Guo, Huimei Li, Yilei Li, Min Weng, Shukuan Wu. **Heilongjiang** Provincial CDC: Shichun Yan, Mingyuan Zou, Xue Zhou. Nangang CDC: Ziyan Guo, Quan Kang, Yanjie Li, Bo Yu, Qinai Xu. **Henan** Provincial CDC: Liang Chang, Lei Fan, Shixian Feng, Ding Zhang, Gang Zhou. Huixian CDC: Yulian Gao, Tianyou He, Pan He, Chen Hu, Huarong Sun, Xukui Zhang. **Hunan** Provincial CDC: Biyun Chen, Zhongxi Fu, Yuelong Huang, Huilin Liu, Qiaohua Xu, Li Yin. Liuyang CDC: Huajun Long, Xin Xu, Hao Zhang, Libo Zhang. **Jiangsu** Provincial CDC: Jian Su, Ran Tao, Ming Wu, Jie Yang, Jinyi Zhou, Yonglin Zhou. Suzhou CDC: Yihe Hu, Yujie Hua, Jianrong Jin Fang Liu, Jingchao Liu, Yan Lu, Liangcai Ma, Aiyu Tang, Jun Zhang. **Qingdao** CDC: Liang Cheng, Ranran Du, Ruqin Gao, Feifei Li, Shanpeng Li, Yongmei Liu, Feng Ning, Zengchang Pang, Xiaohui Sun, Xiaocao Tian, Shaojie Wang, Yaoming Zhai, Hua Zhang, Licang CDC: Wei Hou, Silu Lv, Junzheng Wang. **Sichuan** Provincial CDC: Xiaofang Chen, Xianping Wu, Ningmei Zhang, Weiwei Zhou. Pengzhou CDC: Xiaofang Chen, Jianguo Li, Jiaqiu Liu, Guojin Luo, Qiang Sun, Xunfu Zhong. **Zhejiang** Provincial CDC: Weiwei Gong, Ruying Hu, Hao Wang,Meng Wang, Min Yu. Tongxiang CDC: Lingli Chen, Qijun Gu, Dongxia Pan，Chunmei Wang, Kaixu Xie, Xiaoyi Zhang.

**Supplementary methods**

*Alcohol intake assessment*

Details of alcohol use were documented by standard questionnaire at baseline and two subsequent resurveys. In the questionnaire, participants were asked about the frequency of drinking during past 12 months (never or almost never, only occasionally, only at certain seasons, every month but less than weekly, and usually at least once a week). Those who did not drank at least once a week were asked whether they ever had a period of at least one year, during which they usually drank some alcohol at least once a week. Those who drank usually at least once a week during past 12 months were further asked about the frequency of drinking in a typical week (1-2 days/week, 3-5 days/week, and daily or almost every day), and alcohol type (beer, rice wine, wine, and spirit) and quantity consumed (in small bottle [250 ml] or large [640 ml] bottles for beer and number of liang [50 g] for other alcohol type) on a typical day, special day, or last time the participant drank.

Based on the questionnaire, participants were categorized into ex-drinkers (had none or occasional [i.e. occasionally, only at certain seasons, every month but less than weekly] alcohol use in the past 12 months but had previously drunk in most weeks), non-drinkers (had never or almost never drunk alcohol in the past 12 months and had not drunk at least weekly in the past), occasional drinkers (had drunk alcohol in the past 12 months only occasionally, at certain seasons, or monthly but less than weekly, and had not drunk at least weekly in the past), and current drinkers (had drunk alcohol at least weekly in the past 12 months) who were further divided into four groups in men (<140, 140-279, 280-419, and ≥420 g/week) and two groups in women (<70 and ≥70 g/week) based on baseline weekly alcohol intake.

To calculate overall mean alcohol intake at baseline, resurvey 1 and resurvey 2 for conventional epidemiological analyses, 5 g/week was allocated to occasional drinkers at the time of survey regardless of past drinking patterns (i.e. occasional drinkers, and ex-drinkers who still drank occasionally at the time of the survey). 0 g/week was allocated to non-drinkers at the time of survey (i.e. non-drinkers, and ex-drinkers who had no alcohol use in the past 12 months). In the calculation of mean alcohol intake in genetic analyses, however, 5 g/week was assigned to occasional drinkers and ex-drinkers were excluded.

*Carotid artery measurements assessment*

Details of carotid artery ultrasonography measurements were described previously (1). The carotid artery measurements were acquired by a Panasonic CardioHealth Station. During carotid ultrasound examination, participants lied in the supine position with head tilted at approximately 45º to the contralateral side while resting their head using a triangular pillow. Longitudinal images of the far vessel wall of the carotid artery were recorded using a high-resolution linear ultrasound probe. Carotid intima media thickness (cIMT) was displayed by B-mode ultrasound as a double line pattern on the far wall of the common carotid artery (CCA). The cIMT was measured in the distal 1cm of the CCA just before the bifurcation at four predefined angles (two on each side), including the right CCA at 150º and 120º and the left CCA at 210º and 240º. Mean cIMT was estimated as the mean of four measurements. The cIMT measurements were recorded by Panasonic device automatically at end-diastole using real-time arterial distension data from the target segment of the CCA.

Longitudinal scanning of the entire length of the carotid arteries (from the base of the neck to the angle of the jaw) was conducted bilaterally to screen for the presence of plaques, including 1 cm distance of CCA, carotid bifurcation, proximal internal carotid artery, and proximal external artery. The number and location of plaques were recorded by the sonographers.

Trained sonographers recorded the measurements and were supervised by one of three radiologists who were experienced in the conduct and interpretation of carotid ultrasound examinations. A one-week training programme including a written protocol and video of the examination procedures provided detailed guidance on the optimum methods to record carotid artery measurements. A random sample (~3%) was selected to derive an overall quality score, based on the quality checking that cIMT had been measured correctly at 4 CCA segments, and that plaques had been located and counted satisfactorily in each of the 10 CCA segments.

*Genotyping and biochemistry measurements*

A population-based sample of 151,035 participants was randomly selected from the whole cohort for genotyping. Additional cases with stroke, CHD and COPD were selected for genotyping as part of nested case control studies of CVD and respiratory disease. *ALDH2***-**rs671 and *ADH1B*-rs1229984 were genotyped in a total of 167,734 participants using custom Illumina Golden Gate® (92,968) or Affymetrix Axiom® arrays (94,000) at BGI (Shenzhen, China). The concordance rate was 99.94% for rs1229984 and 99.97% for rs671 in about 25,400 participants who were genotyped by both methods.

Non-fasting blood samples collected at the resurvey were assayed using on-site analysers for lipid measurements (total cholesterol [TC], low-density lipoprotein cholesterol [LDL-C], and high-density lipoprotein cholesterol [HDL-C]; ACON Mission Cholesterol), and glucose (ACON On Call Advanced). Data were available in 19,256 individuals for TC, 17,433 for LDL-C, 20,536 for HDL-C, and 22,150 for random glucose, in the final study population of the present study (n=22,384; **Figure S1**).

**Supplementary reference**

1. Clarke R, Du H, Kurmi O, Parish S, Yang M, Arnold M, Guo Y, Bian Z, Wang L, Chen Y, et al. Burden of carotid artery atherosclerosis in Chinese adults: Implications for future risk of cardiovascular diseases. European Journal of Preventive Cardiology. 2017;24(6):647-56.

**Figure S1. Participant flow chart**

512,726 participants in China Kadoorie Biobank

22,384 participants with complete data for alcohol details, both genetic variants (rs671 G>A, rs1229984 G>A), and all three outcomes (cIMT, presence of carotid plaque, and carotid plaque burden)

Excluded those without resurvey alcohol details (n=198), genotyping data (n=2437), and carotid ultrasound measurements (n=220)

25,239 participants attended resurvey between 2013-2014

**Table S1. Self-reported alcohol intake at baseline and resurveys by baseline defined alcohol intake groups, in men and women**

|  |  |  | **Mean alcohol intake, g/week** | | | | | | |
| --- | --- | --- | --- | --- | --- | --- | --- | --- | --- |
|  | **N (%)** |  | **Baseline**  **(2004-2008)** |  | **1^st^ resurvey**  **(2008)** |  | **2^nd^ resurvey**  **(2013-2014)** |  | **Usual intake (g/week)** |
| **Men** |  |  |  |  |  |  |  |  |  |
| Ex-drinkers | 395 (7.9) |  | 2.8 |  | 36.6 |  | 70.6 |  | 53.6 |
| Non-drinkers | 960 (19.2) |  | 0 |  | 3.6 |  | 5.7 |  | 4.7 |
| Occasional drinkers | 1968 (39.5) |  | 5 |  | 19.9 |  | 36.0 |  | 28.0 |
| <140 g/week | 616 (12.4) |  | 78.3 |  | 97.2 |  | 121.9 |  | 109.6 |
| 140-279 g/week | 429 (8.6) |  | 223.0 |  | 183.8 |  | 232.9 |  | 208.4 |
| 280-419 g/week | 290 (5.8) |  | 365.6 |  | 294.9 |  | 291.3 |  | 293.1 |
| 420+ g/week | 328 (6.6) |  | 689.4 |  | 438.2 |  | 428.7 |  | 433.4 |
| **Women** |  |  |  |  |  |  |  |  |  |
| Ex-drinkers | 75 (0.9) |  | 2.2 |  | 13.8 |  | 17.3 |  | 15.6 |
| Non-drinkers | 5260 (63.7) |  | 0 |  | 0.8 |  | 0.7 |  | 0.7 |
| Occasional drinkers | 2752 (33.3) |  | 5 |  | 4.2 |  | 3.5 |  | 3.8 |
| <70 g/week | 76 (0.9) |  | 33.2 |  | 24.8 |  | 21.7 |  | 23.3 |
| 70+ g/week | 96 (1.2) |  | 227.2 |  | 141.2 |  | 115.9 |  | 128.6 |

Calculations were based on a subset of the present study population who completed baseline and the two resurveys. Details on alcohol intake were obtained by the same questionnaires at baseline and the two resurveys. An intake of 5 g/week was assigned to those who drank sometimes but less than weekly at the time of survey, regardless of past drinking patterns. Usual alcohol intake was estimated by taking the average of mean alcohol intake of the two resurveys.

**Table S2. Characteristics of men and women at resurvey**

|  | **Overall**  **n=22384** | **Men**  **n=8503** | **Women**  **n=13881** |  |  |  |
| --- | --- | --- | --- | --- | --- | --- |
|  |  |  |  |  |  |  |
| Mean age, years (SD) | 59.6 (10.2) | 60.3 (10.4) | 59.1 (10.0) |  |  |  |
| Urban residents, % | 43.5 | 43.2 | 43.7 |  |  |  |
| Baseline alcohol intake status, % |  |  |  |  |  |  |
| Non-drinkers | 47.7 | 19.5 | 65.0 |  |  |  |
| Ex-drinkers | 3.6 | 8.0 | 0.9 |  |  |  |
| Occasional drinkers | 34.4 | 38.4 | 32.0 |  |  |  |
| Current drinkers | 14.3 | 34.2 | 2.1 |  |  |  |
| Resurvey alcohol intake status, % |  |  |  |  |  |  |
| Non-drinkers | 64.3 | 35.0 | 82.2 |  |  |  |
| Ex-drinkers | 4.6 | 10.1 | 1.3 |  |  |  |
| Occasional drinkers | 19.2 | 26.4 | 14.7 |  |  |  |
| Current drinkers | 11.9 | 28.5 | 1.8 |  |  |  |
| Education ≥ 6 years, % | 47.5 | 57.3 | 41.5 |  |  |  |
| Household income ≥ 35000 RMB/year, % | 60.0 | 62.6 | 58.3 |  |  |  |
| Current smokers, % | 20.1 | 50.5 | 1.5 |  |  |  |
| Previous CVD at baseline, % | 3.7 | 3.8 | 3.6 |  |  |  |
| Previous CVD at resurvey, % | 8.8 | 9.3 | 8.5 |  |  |  |
| Mean SBP, mmHg (SD) | 136.7 (20.7) | 137.0 (19.7) | 136.5 (21.3) |  |  |  |
| Mean BMI, kg/m^2^ (SD) | 24.2 (3.5) | 24.0 (3.4) | 24.3 (3.5) |  |  |  |
| Mean total cholesterol, mmol/L (SD) | 4.31 (1.03) | 4.14 (0.93) | 4.41 (1.07) |  |  |  |
| Mean LDL-C, mmol/L (SD) | 2.19 (0.91) | 2.16 (0.87) | 2.20 (0.93) |  |  |  |
| Mean HDL-C, mmol/L (SD) | 1.38 (0.42) | 1.28 (0.42) | 1.43 (0.41) |  |  |  |
| Mean random glucose, mmol/L (SD) | 5.86 (2.15) | 5.74 (2.14) | 5.94 (2.15) |  |  |  |
| Physical activity, mean MET-h/day (SD) | 18.1 (13.7) | 18.8 (15.0) | 17.6 (12.8) |  |  |  |
| cIMT, mm (SD) | 0.67 (0.15) | 0.70 (0.16) | 0.64 (0.13) |  |  |  |
| Carotid plaque, % | 31.3 | 39.1 | 26.5 |  |  |  |
| Carotid plaque burden, mm (SD) | 0.78 (1.08) | 0.99 (1.19) | 0.65 (0.99) |  |  |  |

7145, 6354, 7833 and 8409 men had measurements of total cholesterol, LDL-C, HDL-C and random glucose, respectively; 12111, 11079, 12703 and 13733 women had measurements of total cholesterol, LDL-C, HDL-C and random glucose, respectively. RMB: renminbi; CVD: cardiovascular disease; SBP: systolic blood pressure; BMI: body mass index; MET: metabolic equivalents of task per hour per day; LDL-C: low-density lipoprotein cholesterol; HDL-C: high-density lipoprotein cholesterol; cIMT: carotid intima media thickness; SD: standard deviation.

**Figure S2. Proportion of current drinkers, and mean alcohol intake among current drinkers, in men and women by study areas**


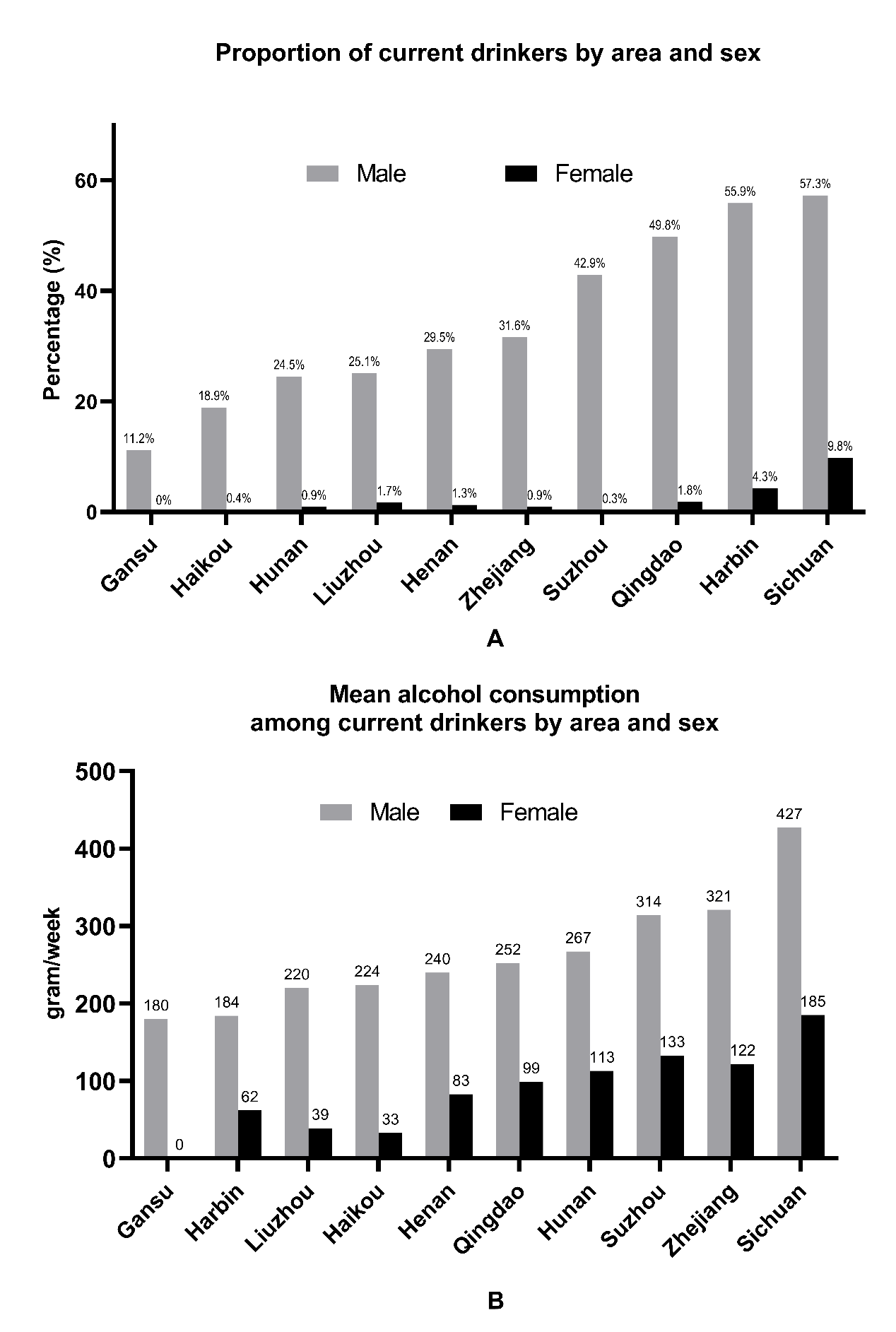


**Table S3. Allele frequency of ADH1B-rs1229984 G>A and ALDH2-rs671 G>A, by study areas**

|  |  | **Overall** | **Qingdao (Urban)** | **Harbin (Urban)** | **Haikou (Urban)** | **Suzhou (Urban)** | **Liuzhou (Urban)** | **Sichuan (Rural)** | **Gansu (Rural)** | **Henan (Rural)** | **Zhejiang (Rural)** | **Hunan (Rural)** |
| --- | --- | --- | --- | --- | --- | --- | --- | --- | --- | --- | --- | --- |
| ***ADH1B-*rs1229984** | N | 22384 | 1433 | 1899 | 1247 | 2586 | 2579 | 2277 | 2220 | 2834 | 2764 | 2545 |
|  | A-allele frequency | 0.21 | 0.18 | 0.16 | 0.28 | 0.23 | 0.25 | 0.20 | 0.15 | 0.13 | 0.29 | 0.27 |
| ***ALDH2-*rs671** | N | 22384 | 1433 | 1899 | 1247 | 2586 | 2579 | 2277 | 2220 | 2834 | 2764 | 2545 |
|  | A-allele frequency | 0.70 | 0.71 | 0.66 | 0.74 | 0.71 | 0.71 | 0.67 | 0.64 | 0.68 | 0.72 | 0.73 |

**Table S4*.* Alcohol drinking patterns by *ALDH2-*rs671 and *ADH1B-*rs1229984 genotypes, in men and women**

|  | ***ALDH2-*rs671** | | |  | ***ADH1B-*rs1229984** | | |  |
| --- | --- | --- | --- | --- | --- | --- | --- | --- |
|  | **AA** | **AG** | **GG** | **P_trend_**^†^ | **AA** | **AG** | **GG** | **P_trend_**^†^ |
| **Men, N** | 428 | 2804 | 5271 |  | 4089 | 3663 | 751 |  |
| Ex-drinkers, % | 1.2 | 5.3 | 10.0 | <0.001 | 7.9 | 8.2 | 7.3 | 0.764 |
| Non-drinkers, % | 74.1 | 29.9 | 9.5 | <0.001 | 20.7 | 18.9 | 15.1 | <0.001 |
| Occasional drinkers, % | 24.4 | 48.3 | 34.2 | <0.001 | 39.2 | 38.4 | 33.8 | 0.009 |
| Current drinkers, % | 0.3 | 16.5 | 46.3 | <0.001 | 32.1 | 34.5 | 43.8 | <0.001 |
| Mean (SD) alcohol intake in current drinkers, g/week^*^ | 32.9  (7.0) | 191.2 (170.0) | 298.2 (243.5) | <0.001 | 270.0 (234.1) | 283.7 (237.0) | 310.5 (240.3) | <0.001 |
| **Women, N** | 664 | 4596 | 8621 |  | 6758 | 5818 | 1305 |  |
| Ex-drinkers, % | 0.1 | 0.5 | 1.2 | 0.046 | 0.8 | 1.0 | 0.8 | 0.766 |
| Non-drinkers, % | 84.5 | 71.9 | 59.8 | <0.001 | 65.8 | 64.5 | 62.7 | 0.004 |
| Occasional drinkers, % | 15.2 | 26.9 | 36.0 | <0.001 | 31.3 | 32.5 | 33.7 | 0.008 |
| Current drinkers, % | 0.2 | 0.6 | 3.1 | <0.001 | 2.1 | 2.0 | 2.9 | 0.990 |
| Mean (SD) alcohol intake in current drinkers, g/week^*^ | -- | 98.2  (76.9) | 133.6 (136.4) | 0.742 | 128.1 (119.2) | 127.8 (136.6) | 144.0 (158.0) | 0.472 |

Proportions and means were adjusted for age at resurvey and study area.

^*^ Calculations assign an intake of 5 g/week to occasional drinkers, excluding ex-drinkers.

^†^ P for trend per G-allele increase is from inverse-variance-weighted meta-analysis across ten areas, adjusted for age at resurvey within areas.

**Table S5. Associations of carotid measurements with self-reported alcohol consumption in men**

|  | **Ex-weekly**  **(n=550)** | **Non-drinkers**  **(n=1460)** | **Occasional**  **(n=2930)** | **Current drinkers** | | | |
| --- | --- | --- | --- | --- | --- | --- | --- |
|  |  |  |  | **<140 g/week**  **(n=944)** | **140-279 g/week**  **(n=705)** | **280-419 g/week**  **(n=475)** | **420+ g/week**  **(n=513)** |
| **cIMT, mm (adjusted mean, 95% CI)** | | | | | | |  |
| Full model | 0.695 (0.684, 0.706) | 0.701 (0.694, 0.708) | 0.694 (0.689, 0.699) | 0.686 (0.678, 0.695) | 0.699 (0.0.689, 0.708) | 0.690 (0.679, 0.702) | 0.688 (0.676, 0.699) |
| Full model + SBP | 0.693 (0.682, 0.704) | 0.703 (0.696, 0.710) | 0.695 (0.691, 0.700) | 0.687 (0.679, 0.696) | 0.696 (0.686, 0.705) | 0.687 (0.675, 0.699) | 0.681 (0.669, 0.693) |
| Full model + SBP and BMI | 0.692 (0.681, 0.703) | 0.703 (0.696, 0.710) | 0.695 (0.691, 0.700) | 0.687 (0.679, 0.695) | 0.695 (0.686, 0.705) | 0.688 (0.676, 0.699) | 0.681 (0.669, 0.692) |
| Full model + SBP, BMI and LDL-C | 0.694 (0.681-0.706) | 0.703 (0.695-0.711) | 0.695 (0.69-0.701) | 0.685 (0.676-0.695) | 0.693 (0.681-0.704) | 0.682 (0.669-0.696) | 0.685 (0.672-0.698) |
| Sensitivity analysis of full model | 0.709 (0.699, 0.720) | 0.712 (0.705, 0.719) | 0.703 (0.698, 0.708) | 0.700 (0.691, 0.708) | 0.710 (0.701, 0.720) | 0.700 (0.688, 0.712) | 0.698 (0.686, 0.710) |
| **Carotid plaque (OR, 95% CI)** | | | | | | |  |
| Full model | 1.24 (1.02, 1.50) | 1.00 (0.88, 1.14) | 0.94 (0.86, 1.03) | 1.00 (0.86, 1.17) | 1.16 (0.98, 1.38) | 1.28 (1.04, 1.58) | 1.50 (1.22, 1.84) |
| Full model + SBP | 1.19 (0.98, 1.44) | 1.00 (0.88, 1.14) | 0.94 (0.86, 1.03) | 0.99 (0.85, 1.16) | 1.10 (0.92, 1.31) | 1.20 (0.97, 1.48) | 1.36 (1.11, 1.68) |
| Full model + SBP and BMI | 1.19 (0.99, 1.45) | 1.00 (0.88, 1.14) | 0.94 (0.86, 1.03) | 0.99 (0.85, 1.16) | 1.10 (0.92, 1.31) | 1.20 (0.97, 1.48) | 1.36 (1.11, 1.68) |
| Full model + SBP, BMI and LDL-C | 1.21 (0.96-1.51) | 1.00 (0.85-1.17) | 0.92 (0.82-1.02) | 0.91 (0.76-1.09) | 1.04 (0.85-1.29) | 1.24 (0.97-1.57) | 1.41 (1.12-1.79) |
| Sensitivity analysis of full model | 1.34 (1.13, 1.59) | 1.00 (0.88, 1.13) | 0.92 (0.84, 1.00) | 1.00 (0.86, 1.15) | 1.16 (0.99, 1.37) | 1.26 (1.03, 1.54) | 1.43 (1.17, 1.74) |
| **Carotid plaque burden, mm (adjusted mean, 95% CI)** | | | | | | |  |
| Full model | 0.96 (0.88, 1.04) | 0.89 (0.84, 0.94) | 0.85 (0.82, 0.89) | 0.85 (0.78, 0.91) | 0.97 (0.90, 1.04) | 1.04 (0.95, 1.13) | 1.06 (0.97, 1.14) |
| Full model + SBP | 0.95 (0.87, 1.03) | 0.90 (0.85, 0.95) | 0.86 (0.82, 0.90) | 0.85 (0.79, 0.91) | 0.95 (0.88, 1.02) | 1.02 (0.93, 1.11) | 1.02 (0.93, 1.10) |
| Full model + SBP and BMI | 0.95 (0.87, 1.03) | 0.90 (0.85, 0.95) | 0.86 (0.83, 0.90) | 0.85 (0.79, 0.91) | 0.95 (0.88, 1.02) | 1.02 (0.93, 1.10) | 1.02 (0.93, 1.10) |
| Full model + SBP, BMI and LDL-C | 0.95 (0.86-1.04) | 0.91 (0.85-0.97) | 0.87 (0.83-0.91) | 0.83 (0.76-0.90) | 0.95 (0.87-1.03) | 1.03 (0.93-1.13) | 1.05 (0.95-1.15) |
| Sensitivity analysis of full model | 1.09 (1.02 1.17) | 0.99 (0.94, 1.04) | 0.93 (0.89, 0.97) | 0.93 (0.86, 0.99) | 1.06 (0.99, 1.13) | 1.12 (1.03, 1.21) | 1.12 (1.03, 1.21) |

The reference group was non-drinkers in all models. Full models were adjusted for age, area, education, income, and smoking. Participants with self-reported history of cardiovascular disease at baseline or resurvey were excluded from full model and were included in the sensitivity analysis. Participants with missing LDL-C or HDL-C data were excluded from models adjusted for LDL-C or/and HDL-C. cIMT: carotid intima media thickness; OR: odds ratio; CI: confidence interval; SBP: systolic blood pressure; BMI: body mass index; LDL-C: low-density lipoprotein cholesterol; HDL-C: high-density lipoprotein cholesterol.

**Table S6. Associations of carotid measurements with self-reported alcohol intake, among subgroups in male current drinkers**

| **Subgroup** | **N** | **cIMT mm, mean difference per 280 g/week (95% CI)** | **P_trend_** | **Carotid plaque, OR per 280 g/week (95% CI)** | **P_trend_** | **Carotid plaque burden mm, mean difference per 280 g/week (95% CI)** | **P_trend_** |
| --- | --- | --- | --- | --- | --- | --- | --- |
| Age, years |  |  |  |  |  |  |  |
| <55 | 2707 | 0.006 (-0.010,0.023) |  | 1.16 (0.73,1.84) |  | 0.07 (-0.04,0.17) |  |
| 55-64 | 2576 | 0.010 (-0.012,0.031) |  | 1.62 (1.15,2.27) |  | 0.30 (0.15,0.46) |  |
| ≥65 | 2294 | -0.023 (-0.049,0.003) | 0.111 | 1.27 (0.87,1.85) | 0.897 | 0.21 (0.01,0.42) | 0.054 |
| LDL-C, mmol/L |  |  |  |  |  |  |  |
| <1.8 | 2058 | 0.009 (-0.011,0.028) |  | 1.86 (1.23,2.81) |  | 0.24 (0.10,0.39) |  |
| 1.8-2.5 | 1993 | -0.020 (-0.042,0.003) |  | 1.66 (1.08,2.57) |  | 0.24 (0.07,0.41) |  |
| ≥2.6 | 1590 | 0.031 (-0.001,0.063) | 0.682 | 1.34 (0.83,2.16) | 0.314 | 0.27 (0.04,0.50) | 0.875 |
| Glucose, mmol/L |  |  |  |  |  |  |  |
| <5 | 2768 | -0.003 (-0.023,0.018) |  | 1.24 (0.82,1.88) |  | 0.15 (0.00,0.31) |  |
| 5-5.9 | 2602 | 0.011 (-0.010,0.032) |  | 1.47 (1.03,2.10) |  | 0.23 (0.07,0.38) |  |
| ≥6 | 2117 | -0.008 (-0.031,0.014) | 0.789 | 1.52 (1.04,2.23) | 0.486 | 0.21 (0.04,0.38) | 0.608 |
| SBP, mmHg |  |  |  |  |  |  |  |
| <120 | 1470 | 0.002 (-0.023,0.027) |  | 0.84 (0.45,1.57) |  | -0.02 (-0.21,0.17) |  |
| 120-139 | 3188 | -0.005 (-0.023,0.013) |  | 1.43 (1.01,2.02) |  | 0.13 (0.00,0.26) |  |
| ≥140 | 2919 | -0.006 (-0.027,0.015) | 0.659 | 1.38 (1.00,1.90) | 0.323 | 0.25 (0.10,0.41) | 0.029 |
| BMI, kg/m^2^ |  |  |  |  |  |  |  |
| <22.5 | 3134 | 0.005 (-0.015,0.026) |  | 1.62 (1.12,2.37) |  | 0.22 (0.06,0.38) |  |
| 22.5-24.9 | 2218 | -0.001 (-0.023,0.021) |  | 1.31 (0.87,1.97) |  | 0.12 (-0.04,0.29) |  |
| ≥25 | 2224 | -0.004 (-0.025,0.016) | 0.519 | 1.36 (0.95,1.94) | 0.505 | 0.23 (0.09,0.37) | 0.852 |

Models were adjusted for age, area, education, household income, and smoking, as appropriate. cIMT: carotid intima media thickness; CI: confidence interval; LDL-C: low density lipoprotein cholesterol; SBP: systolic blood pressure; BMI: body mass index

**Table S7. Associations of carotid measurements with self-reported alcohol consumption in women**

|  | **Ex-drinkers**  **(n=104)** | **Non-drinkers**  **(n=8133)** | **Occasional**  **(n=3973)** | **Current drinkers** | |
| --- | --- | --- | --- | --- | --- |
|  |  |  |  | **<70 g/week**  **(n=145)** | **70+ g/week**  **(n=115)** |
| cIMT (adjusted mean, 95% CI, mm) | 0.606 (0.586, 0.627) | 0.636 (0.634, 0.638) | 0.633 (0.629, 0.636) | 0.633 (0.614, 0.653) | 0.621 (0.603, 0.639) |
| Carotid plaque (OR, 95% CI) | 0.72 (0.44, 1.18) | 1.00 (0.93, 1.08) | 0.94 (0.86, 1.02) | 0.57 (0.35, 0.95) | 1.22 (0.80, 1.86) |
| Carotid plaque burden (adjusted mean, 95% CI, mm) | 0.35 (0.19, 0.50) | 0.60 (0.59, 0.62) | 0.57 (0.54, 0.60) | 0.46 (0.31, 0.61) | 0.61 (0.47, 0.74) |

Models were adjusted for age, area, education and income level, and smoking status. Reference group: non-drinkers. cIMT: carotid intima media thickness; CI: confidence interval; OR: odds ratio.

**Table S8. Associations of carotid measurements with genotype-predicted mean alcohol intake, in men**

|  | **Genotype-predicted mean male alcohol intake categories** | | | | | | **Effect per 280 g/week mean male alcohol intake** | **P-value** |
| --- | --- | --- | --- | --- | --- | --- | --- | --- |
|  | **C1**  **(n=620)** | **C2**  **(n=991)** | **C3**  **(n=1652)** | **C4**  **(n=2016)** | **C5**  **(n=1209)** | **C6**  **(n=2015)** |  |  |
|  |  |  |  |  |  |  |  |  |
| **cIMT, mm (mean, 95% CI)** | | | | | | |  |  |
| Full model | 0.702 (0.691, 0.713) | 0.708 (0.697, 0.718) | 0.709 (0.701, 0.716) | 0.704 (0.696, 0.712) | 0.715 (0.705, 0.725) | 0.697 (0.689, 0.704) | -0.008 (-0.018, 0.003) | 0.148 |
| Sensitivity analysis | 0.695 (0.684, 0.706) | 0.697 (0.687, 0.708) | 0.700 (0.692, 0.707) | 0.694 (0.686, 0.702) | 0.701 (0.690, 0.712) | 0.685 (0.677, 0.692) | -0.010 (-0.020, 0.0005) | 0.061 |
| **Carotid plaque (OR, 95% CI)** | | | | | | |  |  |
| Full model | 1.00 (0.83, 1.21) | 1.05 (0.88, 1.24) | 1.10 (0.96, 1.26) | 1.05 (0.91, 1.21) | 1.30 (1.08, 1.56) | 1.17 (1.01, 1.35) | 1.21 (0.99, 1.49) | 0.061 |
| Sensitivity analysis | 1.00 (0.82, 1.22) | 0.98 (0.81, 1.17) | 1.09 (0.95, 1.26) | 1.01 (0.87, 1.18) | 1.17 (0.95, 1.43) | 1.13 (0.97, 1.31) | 1.18 (0.95, 1.46) | 0.127 |
| **Carotid plaque burden, mm (mean, 95% CI)** | | | | | | |  |  |
| Full model | 0.92 (0.84, 1.01) | 0.92 (0.85, 1.00) | 1.00 (0.94, 1.05) | 0.97 (0.91, 1.02) | 1.07 (0.99, 1.14) | 1.02 (0.96, 1.07) | 0.09 (0.02, 0.17) | 0.018 |
| Sensitivity analysis | 0.86 (0.78, 0.94) | 0.83 (0.75, 0.90) | 0.92 (0.86, 0.98) | 0.87 (0.81, 0.93) | 0.95 (0.87, 1.03) | 0.94 (0.89, 1.00) | 0.09 (0.01, 0.16) | 0.024 |

The category with the lowest mean alcohol intake was the reference group. Models were adjusted for age, area, and genomic principal components. Participants with prior cardiovascular disease at baseline or resurvey were included in full models and excluded in sensitivity analysis. The mean change or OR (95% CI) per 280 g/week genotype-predicted mean male alcohol intake was obtained within each study area and combined by inverse-variance-weighted meta-analysis. cIMT: carotid intima media thickness; CI: confidence interval; OR: odds ratio.

**Table S9. Associations of carotid measurements with six genotypic categories, in women**

|  | **Genotypic categories** | | | | | | **Effect per 280 g/week MEAN MALE alcohol intake** | **P-value** |
| --- | --- | --- | --- | --- | --- | --- | --- | --- |
|  | **C1**  **(n=1036)** | **C2**  **(n=1636)** | **C3**  **(n=2557)** | **C4**  **(n=3295)** | **C5**  **(n=1703)** | **C6**  **(n=3654)** |  |  |
|  |  |  |  |  |  |  |  |  |
| cIMT, mm  (adjusted mean, 95% CI) | 0.645 (0.638, 0.652) | 0.641 (0.635, 0.647) | 0.648 (0.643, 0.652) | 0.645 (0.640, 0.649) | 0.639 (0.633, 0.646) | 0.643 (0.639, 0.648) | -0.004 (-0.011, 0.002) | 0.186 |
| Carotid plaque  (OR, 95% CI) | 1.00 (0.84, 1.19) | 1.07 (0.93, 1.24) | 1.31 (1.15, 1.49) | 1.05 (0.93, 1.17) | 1.13 (0.96, 1.33) | 1.16 (1.02, 1.32) | 0.98 (0.81, 1.17) | 0.806 |
| Carotid plaque burden, mm  (adjusted mean, 95% CI) | 0.66 (0.61, 0.71) | 0.63 (0.58, 0.68) | 0.69 (0.65, 0.73) | 0.63 (0.59, 0.66) | 0.66 (0.61, 0.71) | 0.65 (0.62, 0.69) | -0.01 (-0.06, 0.03) | 0.600 |

The category with the lowest mean alcohol intake was the reference group. Models were adjusted for age, area, and genomic principal components. The mean change or OR (95% CI) per 280 g/week genotype-predicted MEAN MALE alcohol intake was obtained within each study area and combined by inverse-variance-weighted meta-analysis. cIMT: carotid intima media thickness; CI: confidence interval; OR: odds ratio.
